# Identification and Functional Characterization of Genes Encoding Phenylacetaldehyde Reductases That Catalyze the Last Step in the Biosynthesis of Hydroxytyrosol in Olive

**DOI:** 10.3390/plants10071268

**Published:** 2021-06-22

**Authors:** Rosario Sánchez, Cristina Bahamonde, Carlos Sanz, Ana G. Pérez

**Affiliations:** Department of Biochemistry and Molecular Biology of Plant Products, Instituto de la Grasa (CSIC), 41013 Sevilla, Spain; rsanchez@ig.csic.es (R.S.); agprduran@gmail.com (C.B.); carlos.sanz@ig.csic.es (C.S.)

**Keywords:** hydroxytyrosol, phenyl acetaldehyde reductase, olive fruit, phenolic compounds, virgin olive oil, *Olea europaea*

## Abstract

Hydroxytyrosol derivatives are the most important phenolic components in virgin olive oil due to their well-demonstrated biological activities. In this regard, two phenyl acetaldehyde reductase genes, *OePAR1.1* and *OePAR1.2,* involved in hydroxytyrosol synthesis, have been identified from an olive transcriptome. Both genes were synthesized and expressed in *Escherichia coli*, and their encoded proteins were purified. The recombinant enzymes display high substrate specificity for 2,4-dihydroxyphenylacetaldehyde (3,4-DHPAA) to form hydroxytyrosol. The reaction catalyzed by OePAR constitutes the second, and last, biochemical step in the formation of hydroxytyrosol from the amino acid L-3,4-dihydroxyphenylalanine (L-DOPA) in olive. OePAR1.1 and OePAR1.2 enzymes exhibit high thermal stability, similar pH optima (pH 6.5), and high affinity for 3,4-DHPAA (apparent *K*m 0.6 and 0.8 µmol min^−1^ mg^−1^, respectively). However, OePAR1.2 exhibited higher specific activity and higher expression levels in all the olive cultivars under study. The expression analyses indicate that both *OePAR1.1* and *OePAR1.2* genes are temporally regulated in a cultivar-dependent manner. The information provided here could be of interest for olive breeding programs searching for new olive genotypes with the capacity to produce oils with higher levels of hydroxytyrosol derivatives.

## 1. Introduction

The most relevant phenolic compounds in olive (*Olea europaea* L.) belong to the secoiridoids family [1]. In the olive fruit, the main phenolic components are the secoiridoid glycosides oleuropein and ligstroside, whose aglycones are esters of the elenolic acid with the phenolic alcohols hydroxytyrosol (3,4-DHPEA) or tyrosol (*p*-HPEA). During the industrial process to obtain virgin olive oil (VOO), the secoiridoid glucosides, initially present in the olive fruit tissues, are hydrolyzed by β-glucosidases into secoiridoid derivatives less hydrophilic than the originals, and therefore, making them partially soluble in the oil matrix [2,3]. These secoiridoid derivatives are mainly the aldehyde and dialdehyde forms of oleuropein and ligstroside aglycones which are by far the major phenolic compounds in VOO [4]. These phenolic components are directly associated to the functional properties [5], as well as to the flavor [6], of this highly appreciated Mediterranean food. The VOO phenolic components containing hydroxytyrosol possess the highest biological activities and are the most effective in the prevention of chronic diseases due to their superior capacity to reduce chronic inflammation and oxidative damage, as well as due to their stronger antiproliferative properties [7,8,9,10]. The scientific evidences on the health benefits of hydroxytyrosol have led the European Union to approve a health claim on olive oil polyphenols which may be applied to those oils containing at least 250 ppm of hydroxytyrosol and its derivatives [11].

In recent years, there has been a growing interest in the hydroxytrosol derivatives as functional components of VOO or other olive derived foods, and also as new functional ingredients to be used in the cosmetic, pharmaceutical, and food industries [12]. It is quite well stablished that the main endogenous factors positively affecting the accumulation of phenolic components in VOO are the content of phenolic glucosides and the level of β-glucosidase activity in the olive fruit [13,14]. On the contrary, polyphenol oxidase and peroxidase, catalyzing the oxidative degradation of secoiridoid compounds, limit the phenolic content of the oils [3]. However, the knowledge of the biochemical processes that give rise to the accumulation of phenolic glucosides in the olive fruit is far from being known [15]. From transcriptomic studies, several genes supposedly involved in the phenolic metabolism of olive fruit have been identified [14,15,16,17], but only in a few cases have any of these genes been functionally characterized and their involvement in the biosynthetic pathways of the phenolic compounds demonstrated [18,19,20]. In this sense, the specific genes controlling the biosynthesis pathway for hydroxytyrosol and tyrosol in olive have not been fully elucidated yet.

**Figure 1:**
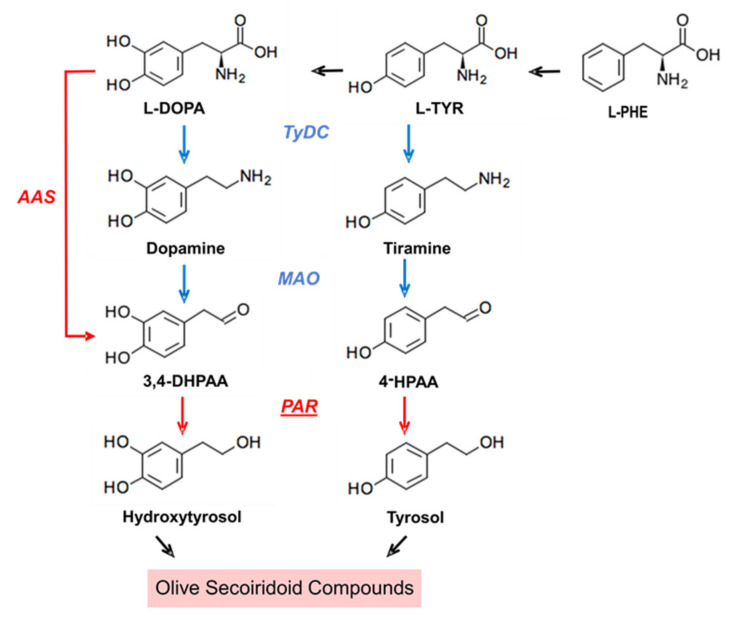
Biosynthesis pathway proposed by Sanchez et al. [26] for the phenolic alcohols hydroxytyrosol and tyrosol which constitute the phenol moiety of secoiridoid compounds in olive. Abbreviated intermediate metabolites and enzymes are: 3,4 dihidroxyphenylacetaldehyde (3,4-DHPAA); 4-hydroxyphenylacetaldehyde (4-HPAA); tyrosine/DOPA decarboxylase (TyDC); monoamino oxidase (MAO); aromatic aldehyde synthase (AAS); phenylacetaldehyde reductase (PAR).

Tyrosol is synthesized in yeast from tyrosine through the well-stablished Ehrlich pathway, that is, amino acids are first transformed into α-ketoacids by transamination, then converted into aldehydes by decarboxylation, and finally reduced to alcohols [21]. Considering this, different strategies for the production of tyrosol and hydroxytyrosol through metabolic engineering have been described. Thus, a procedure for the production of hydroxytyrosol from L-3,4-dihydroxyphenylalanine (L-DOPA) using engineered *Escherichia coli* has been described [22]. Moreover, the overproduction of hydroxytyrosol in *Saccharomyces cerevisiae* by heterologous expression of a 4-hydroxyphenylacetate 3-monooxygenase was reported last year as a way to increase hydroxytyrosol content in wine [23]. The biosynthetic pathway for the production of tyrosol has been studied also in plants. Lan et al. [24] described the generation of tyrosol from tyrosine in *Rhodiola crenulata* by the sequential activity of three discrete enzymes, tyrosine decarboxylase (TyDC; EC 4.1.1.25), monoamine oxidase (MAO; EC 1.4.3.4), and 4-hydroxyphenylacetaldehyde reductase (4HPAR; aryl-alcohol dehydrogenase EC1.1.1.90). A few years later, Torrens-Spence et al. [25] reported that besides the previously mentioned pathway, involving separate decarboxylation and deamination enzymatic steps, the plants of the *Rhodiola* genus contain a pyridoxal phosphate (PLP)-dependent aromatic aldehyde synthase (AAS) that directly converts tyrosine into 4-hydroxyphenylacetaldehyde (4-HPAA). In a recent study, we have identified in olive an aromatic aldehyde synthase gene (*OeAAS*), which was synthesized and expressed in *E. coli* [26]. The encoded protein OeAAS is a bifunctional enzyme catalyzing decarboxylation and amine-oxidation reactions in a single step which displays strict substrate specificity for L-DOPA to form 3,4-dihydroxyphenylacetaldehyde (3,4-DHPAA), the immediate precursor of hydroxytyrosol. The last step in this biosynthesis pathway for the conversion of L-DOPA into hydroxytyrosol would be catalyzed by a specific alcohol dehydrogenase (Figure 1).

Similar enzymes, catalyzing the reduction of phenylacetaldehyde to the volatile compound phenyl ethanol, named as phenyl acetaldehyde reductases (PARs), have been identified in *Solanum lycopersicum* [27], *Rosa damascena* [28], and *Rhodiola rosea* [25]. PAR proteins belong to a large family of NADPH-dependent reductases, which contains homologous enzymes widely distributed in other plant-specialized metabolic pathways, such as the anabolism of sporopollenin, lignin, flavonoid as well as the catabolism of abcisic acid and brassinosteroids [25].

In the framework of the characterization of the genes and enzymes responsible for the key pathways related to phenolic metabolism in olive, the aim of the present work is to study the final metabolic step in the biosynthesis of hydroxytyrosol, that is, the reduction of 3,4-DHPAA into hydroxytyrosol. Two PARs genes (*OePAR1.1* and *OePAR1.2*) have been identified from an olive transcriptome obtained from olive fruits from seven olive cultivars with contrasting phenolic contents. The expression data of the *OePAR1.1* and *OePAR1.2* genes in different cultivars and ripening stages and the catalytic properties of their encoded proteins provide relevant information to understand the biosynthesis of hydroxytyrosol and its related compounds in olive.

## 2. Results and Discussion

### 2.1. Identification and Molecular Characterization of PAR Genes in Olive

The search for sequences homologous [29] to the enzymes Rr4HPAR1 (GenBank accession number MF674524) and Rr4HPAR2 (GenBank accession number MF674525) of *Rhodiola rosea* [25] using the olive genomic tools previously generated [26] showed 24 possible proteins with PAR activity. All of them were annotated as cinnamyl-CoA reductases, but these annotations are frequently incorrect and must be validated. Thus, Kim et al. [30] reported that many Arabidopsis genes annotated as putative cinnamyl alcohol dehydrogenases actually encoded proteins with widely varying substrate specificities. Three of those olive transcripts putatively annotated as cinnamyl-CoA reductases (olive genome database https://denovo.cnag.cat/olive_data (access on 27 November 2020), OE6.OLIVEFAT accession numbers: OE6A039209, OE6A025790 and OE6A018558) showed homology to Rr4HPAR1 with high percentages of identity (88–90%), and high statistical significance (Table S1). Three other transcripts (OE6.OLIVEFAT accession numbers: OE6A068874, OE6A030751 and OE6A074153P) also showed high homology to Rr4HPAR2 (identity percentages from 83% to 79%) and low *e*-values (Table S1), but were discarded as candidate genes to control the final step of tyrosol and hydroxytyrosol biosynthesis since, according to Torrens-Spence et al. [25], the Rr4HPAR2 enzyme has very low activity toward hydroxyphenyl aldehydes as substrate, such as the 4-HPAA, compared to Rr4HPAR1. Besides, these Rr4HPAR2 transcript homologs showed to have very low expression levels compared to the Rr4HPAR1 homologs in the olive transcriptome previously generated for seven representative cultivars (Table S1). Among the three Rr4HPAR1 transcript homologs, those showing the highest expression levels in the olive transcriptome and the highest homology, OE6A039209 (90.0%) and OE6A025790 (88.7%), were selected for molecular and biochemical characterization and named respectively, *OePAR1.1* (GenBank Accession number MW038826) and *OePAR1.2* (GenBank Accession number MW038827). In this sense, Guodong et al. [17] recently assigned to the OE6A025790 gene a key role in polyphenol biosynthesis in a transcriptomic analysis in the Leccino olive cultivar. *OePAR1.1* and *OePAR1.2* are 1287 and 1829-bp long genes containing coding regions of 987 and 981 bp, respectively. The predicted proteins exhibited quite similar lengths (328 and 326 amino acids) and molecular weights (36.1 and 35.7 kDa), but slightly different isoelectric points, 7.59 and 6.97 for OePAR1.1 and OePAR1.2, respectively. The subcellular localization predictor program [31] indicated for both OePARs a greater probability of cytoplasmic localization and soluble character, which was confirmed by the absence of signal peptide [32].

The deduced amino acid sequences of the six transcripts initially selected among the cinnamyl-CoA reductases annotated in the olive transcriptome and those PARs proteins previously characterized from *Rhodiola rosea* [25], *Populus trichocarpa* [33], and *Solanum lycopersicum* [27] were aligned together (Figure S1). A commonly used sequence-based classification for alcohol dehydrogenases [28] stablishes three super families with different sizes of the protein chain: short (SDR, approx. 250 aa), medium (MDR, aprox. 350 aa), and long chain dehydrogenases/reductases (LDR 360–550 aa). According to the NCBI conserved domain search, both OePAR proteins could be classified within the extended SDR (short-chain dehydrogenase/reductase) family. Proteins from this family, in addition to the Rossmann fold (alpha/beta folding pattern with a central beta-sheet) core region typical of all SDRs, have a less conserved C-terminal extension of approximately 100 amino acids. These data are in good agreement with the length of both proteins (326–328 amino acids) that exceeds the usual range of 250 amino acids typical of the SDR family [30]. On the other hand, the alignment showed that OePAR1.1 and OePAR1.2 contain a YXX(S)K motif, a highly conserved active-site pattern within the PAR family [28]. In addition to the Tyr (Y) and Lys (K), OePARs showed an upstream Ser (S) contributing to the active site that forms a canonical catalytical triad in the SDR family. Both olive proteins also include a NADP-binding motif similar to TGXXGXX (GA), a glycine-rich region playing a key role in domain stability that allows access to the NAD(P) pyrophosphate.

**Figure 2:**
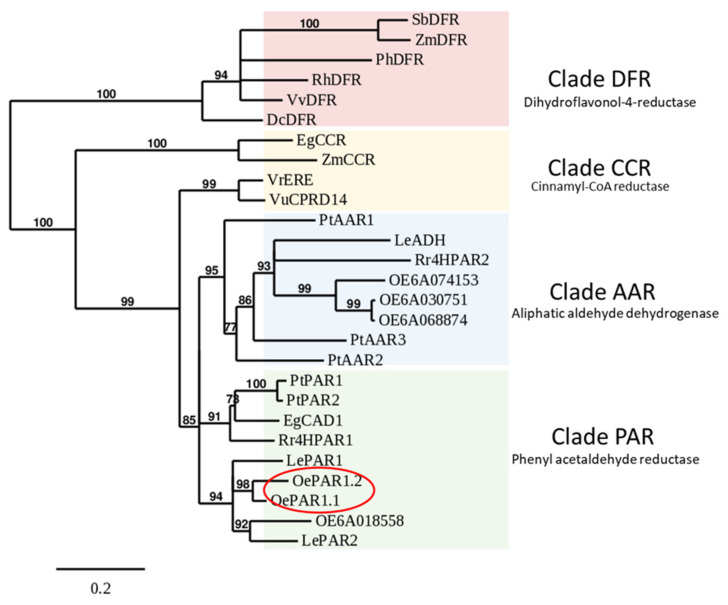
Relationship between *OePAR1.1* and *OePAR1.2* and other phenyl acetaldehyde reductases and related alcohol dehydrogenases of known function. Phylogenetic tree performed on the Phylogeny.fr platform as described in Material and Methods section. Accession numbers are given in Table S2.

A phylogenetic analysis was carried out with the sequences of most PARs characterized so far in plants and diverse sequences of alcohol dehydrogenases and reductases of known function within the extended ADH family (Figure 2). The analysis showed that both proteins are closer to tomato PARs [27] than to RrHPAR1 from *Rhodiola* which was used as a probe. As shown, RrHPAR2, which exhibited extremely low activity toward 4-HPAA [25], clusters with LeADH and PtAAR1 that are only active toward aliphatic aldehydes. These results would sustain that, as previously mentioned, the transcripts OE6A068874, OE6A030751, and OE6A074153 having high homology to RrHPAR2 are probably aliphatic reductases not involved in the biosynthesis of phenolic alcohols.

Given the high homology and the presence of the critical amino acids in the predicted catalytic center, the two candidate genes, OePAR1.1 and OePAR1.2, were regarded as encoding PARs and their corresponding cDNAs were used for recombinant protein production for further characterization.

### 2.2. Catalytic Properties of the Recombinant PAR Proteins

To verify the functional identity of the *OePAR* genes, we cloned the full-length open reading frames corresponding to *OePAR1.1* and *OePAR1.2* into *E. coli* (strain BL21(DE3) lacIq) and expressed them as described in the Material and Methods section. In order to improve their solubility, the culture conditions were adapted for a slow growth (22 h) at a low temperature (19 °C) in LB medium supplemented with NaCl to 0.5 M. The OePAR proteins produced under these conditions were detected in the soluble fractions of *E. coli* cells as a very significant band of the expected size when analyzed by SDS-PAGE, around 37 kDa (Figure S2). Considering the weight of the six histidines added to each construction, the molecular weights calculated from SDS-PAGE are very similar to the theoretical values predicted from the amino acid sequences. Both values are in the same range of those reported for similar enzymes, such as those PARs from tomato [27], rose [28], and camelia [34].

**Figure 3:**
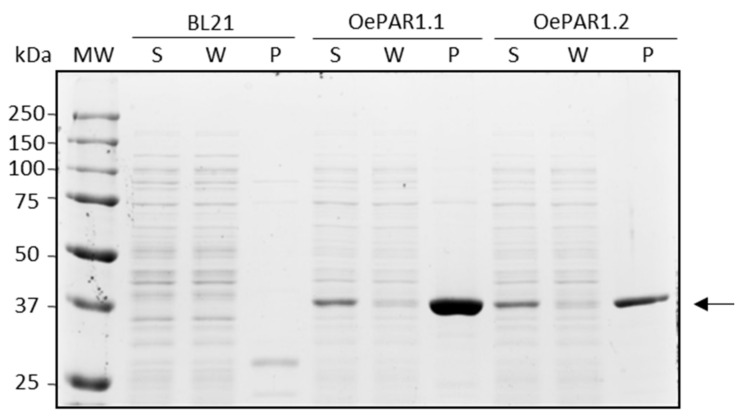
SDS-PAGE followed by a Coomassie blue staining showing recombinant OePAR purification yield by Ni-NTA affinity chromatography compared to the same extracts from untransformed *E. coli* strain BL21 (DE3) LaqIq. The arrow shows OePAR purified (P) proteins from soluble (S) *E. coli* fraction. W: wash fraction; MW: molecular weight ladder.

The purification yield obtained after affinity chromatography was verified by SDS-PAGE electrophoresis of the soluble (S), wash (W), and purified (P) fractions. Figure 3 shows an almost unique band, 98% pure, of approximately 36 kDa in the P fraction for each construction, but absent in the control lane of the untransformed BL21 (DE3) LaqIq. The purified recombinant OePARSs were evaluated for their ability to catalyze the reduction of the phenyl aldehydes 4-HPAA and 3,4-DHPAA to form, respectively, the phenyl alcohols tyrosol and hydroxytyrosol.

**Figure 4:**
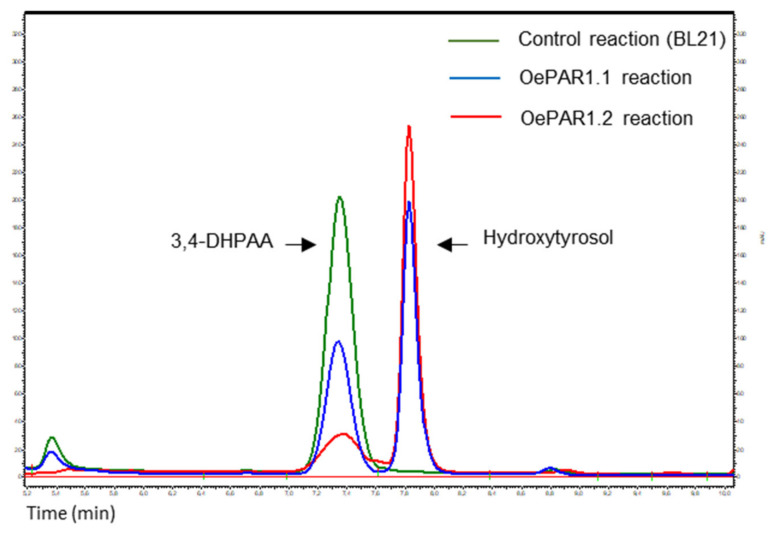
Conversion of 3,4-DHPAA to hydroxytyrosol by the recombinant proteins OePAR 1.1 and OePAR 1.2. Products were analyzed by HPLC and control assay was carried out with BL21 protein extract.

Analysis of the reaction products was carried out by HPLC-DAD/MS as described in Material and Methods section. Results showed that both recombinant proteins, OePAR1.1 and OePAR1.2, displayed strict substrate specificity for 3,4-DHPAA forming hydroxytyrosol as the only reaction product (Figure 4). Besides, both PAR proteins required NADPH as the reducing cofactor. The preference for NADPH instead of NADH has been previously described for other plant PAR proteins. Thus, no activity was detected with LePAR1 and LePAR2 from tomato and Pt PAR1 and Pt PAR2 from poplar when NADH was used as a cofactor [27,33] and only trace activity levels were detected with the rose PAR protein [28].

In vitro activity assays with both proteins using 4-HPAA as a substrate did not result apparently in the formation of tyrosol as a reaction product. The existence of minimal levels of activity with 4-HPAA that could be detectable under other experimental conditions cannot be ruled out. These minimum levels of activity would justify the significant presence of tyrosol derivatives in the fruits, whose average content is approximately ten times lower than the content of hydroxytyrosol derivatives (Table S3). The findings of a very high specificity of OePARs for the formation of hydroxytyrosol support the existence of a pathway for the formation of hydroxytyrosol directly from L-DOPA that we recently proposed [26], which includes an intermediate step mediated by the enzyme aromatic aldehyde synthase, OeAAS, also very specific for the formation of the aldehyde 3,4-DHPAA, so that tyrosol and hydroxytyrosol could have separate synthetic pathways in olive. In this sense, Saimaru and Orihara [35] already suggested that the main biosynthetic pathway of hydroxytyrosol in olive would proceed from L-DOPA through dopamine, while other less effective pathways may be present, like the one converting tyrosol, previously formed from tyramine and 4-HPAA, into hydroxytyrosol [16].

Most of the PARs proteins characterized in plants appear to be highly specific for certain substrates, such as tomato LePAR1 [27] or poplar Pt PAR 1 and Pt PAR2 [33] that are only active toward phenyl acetaldehyde. However, there are some other PAR proteins such as tomato LePAR2 or rose PAR that exhibit moderate non-specific activity with different substrates such as cinnamaldehyde, benzaldehyde, or 2-phenyl propionaldehyde [27,28]. Both olive proteins, OePAR1.1 and OePAR1.2, clearly belong to the first group mentioned. Curiously, most of the enzymes having high substrate specificity appear to be included in the same clade of the phylogenetic tree (Figure 2).

The studies to determine the optimum pH of the two recombinant olive PAR proteins were carried out in a pH range of 4.7–8.4. Both proteins showed maximum activity at pH 6.5 (Figure S3). At this optimum pH, the purified OePAR1.1 and OePAR1.2 proteins exhibited a specific activity of 25.53 and 56.71 µmol min^−1^ mg^−1^, respectively. OePAR1.2 showed a more abrupt drop in specific activity at acidic pH than OePAR1.2, while a very similar trend was observed for both of them at basic pH values. This optimum pH is very similar to that observed for the enzyme that catalyzes the preceding enzymatic step in the pathway, the enzyme OeAAS, which forms 3,4-DHPAA, and which shows a broad pH spectrum of enzymatic activity with a maximum of activity at pH 6.8 [26].

Enzymatic activity tests were carried out in a wide range of temperatures (24–70 °C) in order to determine the optimum temperature for both PAR proteins. Both proteins had maximum activity at 60 °C (Figure S4). However, OePAR1.1 catalytic activity seemed to be more affected by temperature with only 40% residual activity at 30 °C while OePAR1.2 retained 80% of activity at this temperature. The thermal inactivation kinetics, calculated as described in Material and Methods section, showed that both proteins were quite thermostable. OePAR1.1 and OePAR1.2 proteins maintained very high activity, 80% and 95% respectively, after one hour at 50 °C and retained also a significant level of activity after 30 min at 60 °C (Figure S5). Both proteins seem to be extremely stable at ambient temperature losing only 30% and 20% of their activity after 10 days at 25 °C. The thermal stability of olive PAR proteins contrasts with that described for the OeAAS enzyme, which catalyzes the previous stage of hydroxytyrosol synthesis, that is quite unstable at temperatures higher than 30 °C [26].

To obtain the kinetic parameters, recombinant olive PARs enzyme activity was measured over a range of concentrations (0.25–8 mM) of 3,4-DHPAA as substrate (Figure S6). Kinetic characterization of the olive PAR proteins revealed that both enzymes followed typical Michaelis–Menten kinetics. An apparent *K*_m_ of 0.6 mM and a *V*_max_ of 34 µmol min^−1^ mg^−1^ were calculated for OePAR1.1 while a *K*_m_ of 0.8 mM and a *V*_max_ of 86.9 µmol min^−1^ mg^−1^ were calculated for OePAR1.2. These *K*_m_ values are rather similar to those reported in tomato for LePAR2, with benzaldehyde and cinnamaldehyde, 5.7 and 1.1 mM respectively, but much higher than that calculated for LePAR1 with phenylacetaldehyde as substrate, 32 µM [27].

### 2.3. Cultivar and Developmental Expression of Olive PAR Genes

**Figure 5:**
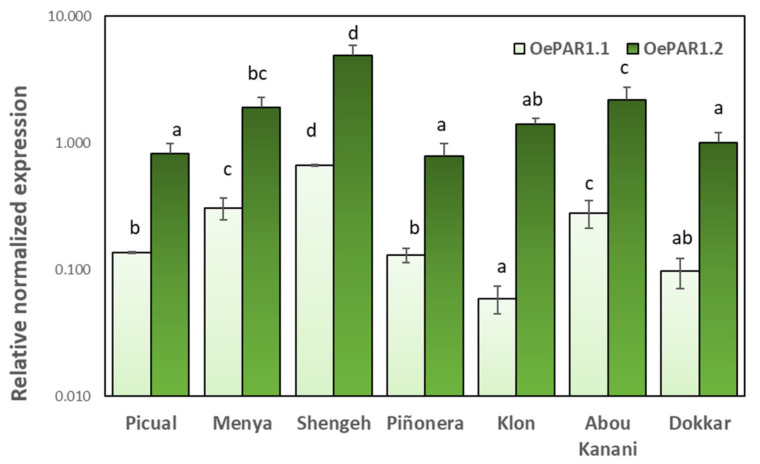
Relative expression levels of olive OePAR1.1 and OePAR1.2 in the mesocarp tissue of Picual, Menya, Shengeh, Piñonera, Klon, Abou kanani, and Dokkar fruits harvested at the commercial maturity stage for olive oil extraction (stage III). The relative expression levels of Oe*PAR* genes were determined using the expression level of the *OePAR1.1* gen from Picual (biological replicate 1) as calibrator. Data are mean ± SD. Three biological and two technical replicates (*n* = 3) were obtained for each cultivar. Different letters indicate statistically significant differences according to Tukey’s test (*p* ≤ 0.05) within each gene.

The expression levels of *OePAR* genes were measured in seven olive cultivars selected from WOGC (IFAPA Alameda del Obispo, Córdoba, Spain) based on their marked differences in terms of phenolic contents [14]. Figure 5 shows the relative expression of both genes in fruits harvested at the commercial maturity stage usually used for VOO extraction (stage III, 28–31 WAF). As shown, the expression levels of *OePAR1.2* were significantly higher than those of *OePAR1.1* in all the cultivars under study.

The highest expression levels for both genes were found in cultivar Shengeh, followed by those in cultivars Abou Kanani and Menya. In our previous study on olive AAS we found that *OeAAS* gene expression was also significantly higher in cultivars Menya and Abou Kanani compared to the other cultivars [26]. However, the highest *OeAAS* expression was found in cultivar Picual, which shows to have low transcription levels for *OePAR1.1* and *OePAR1.2* genes. The lowest expression levels of *OePAR1.1* were found in cultivar Klon, while the cultivars Piñonera, Picual, and Dokkar showed the lowest levels of transcription of *OePAR1.2*. But these cultivars produce oils with contrasting phenolic contents, so that there is no apparent relationship between levels of expression of *OePAR* genes and the levels of final metabolites [26].

**Figure 6:**
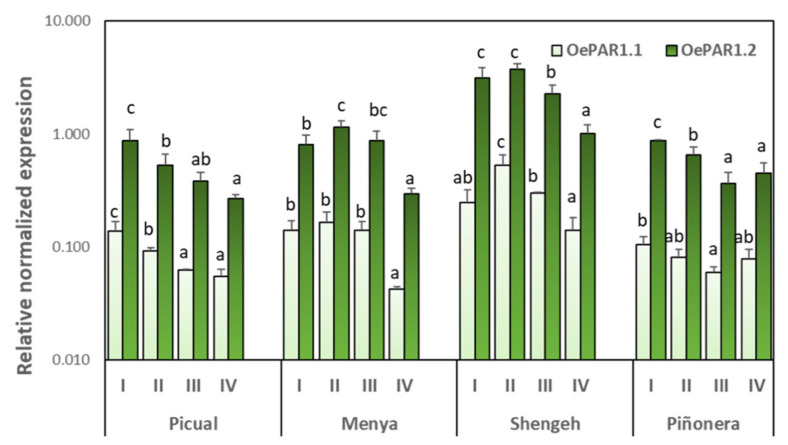
Relative expression levels of olive OePAR1.1 and OePAR1.2 in the mesocarp tissue of olive fruits (cultivars Picual, Menya, Shengeh, Piñonera) at four ripening stages: I (green); II (yellow-green); III (turning stage); and IV (fully ripe). The relative expression levels of Oe*PAR* genes were determined using the expression level of the *OePAR1.1* gene from Picual at ripening stage I (biological replicate 1) as calibrator. Data are mean ± SD. Three biological and two technical replicates (*n* = 3) were obtained for each cultivar and maturity stage. Different letters indicate statistically significant differences according to Tukey’s test (*p* ≤ 0.05) within each cultivar and gene.

In order further study the role of *OePAR1.1* and *OePAR1.2* genes in determining the phenolic composition of VOO, two cultivars producing oils with high phenolic contents, Picual and Menya, and two olive cultivars producing oils with medium/low phenolic contents, Piñonera and Shengeh, were selected (Table S3). The expression levels of *OePAR1.1* and *OePAR1.2* genes (Figure 6) and the phenolic contents of the fruits (Table S4) were analyzed at different ripening stages. Both genes showed similar expression patterns, but significantly lower expression levels were always detected for *OePAR 1.1*. The results demonstrate that *OePARs* expression is temporarily regulated and coincide with that reported for *OeAAS* gene [26]. In the four cultivars, the maximum transcription levels for both genes were observed in stage I (17–19 WAF) and stage II (22–25 WAF), followed by a subsequent decrease in gene expression along ripening (Figure 6). Tieman et al. [27] reported a similar pattern in tomato fruit, with maximum *LePAR1* and *LePAR2* gene expression in mature green fruits and the lowest in red ripe fruits.

**Figure 7:**
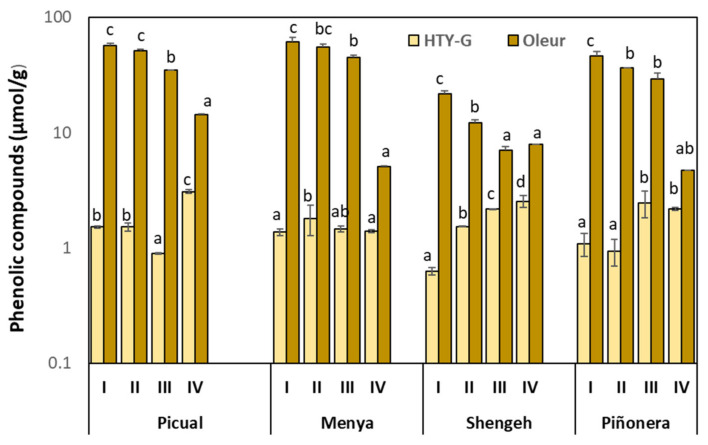
Content of hydroxytyrosol glucoside (HTY-G) and oleuropein (Oleur) in the mesocarp tissue of olive fruits (cultivars Picual, Menya, Shengeh, Piñonera) at four ripening stages: I (green); II (yellow-green); III (turning stage); and IV (fully ripe). Data are mean ± SD (*n* = 3). Different letters indicate statistically significant differences according to Tukey’s test (*p* ≤ 0.05) within each cultivar and metabolite.

The correlation analysis in the four cultivars and four ripening stages showed that there is no relationship between the fruit phenolic content and the expression levels of *OePAR1.1* and *OePAR1.2* genes. More specifically, although this relationship exists within each cultivar throughout the ripening process, negative correlation coefficients were found between the expression of both *OePAR* genes (Figure 6) and the content of the two most significant hydroxytyrosol derivatives in the olive fruit, oleuropein and hydroxytyrosol glucoside (Figure 7), when the values for the four olive cultivars are considered. Thus, Shengeh fruits showed to have the highest *OePAR* transcription levels but the lowest contents of hydroxytyrosol derivatives. Similarly, Tieman et al. [27] did not find in tomato a clear correlation between the expression of *PAR* genes and the accumulation of their catalysis product, mainly phenyl ethanol. Furthermore, the expression analyses carried out in tomato suggest that the limiting step in the synthesis of phenyl alcohols is the formation of the phenyl aldehydes that serve as substrate for PAR proteins. However, transgenic tomato lines with reduced *LePAR* expression did not have reduced 2-phenylethanol levels, suggesting the potential for redundancy in this gene family [27]. In a similar way, divergent trends were found for the expression levels of *RmPAR* gene and the relative content of 2-phenyl ethanol produced in *Rosa moschata* [36]. Considering the positive correlation (*r* = 0.63) found between *OeAAS* expression levels and olive fruit phenolic content in a previous study [26], it seems reasonable to assume that the limiting step in hydroxytyrosol synthesis in olive is also the formation of the phenyl acetaldehyde 3,4-DHPAA, the substrate for the PAR proteins in olive.

## 3. Materials and Methods

### 3.1. Plant Material

Seven olive cultivars (*Olea europaea* L.) from the Core-36 olive nuclear collection maintained at the World Olive Germplasm Collection (WOGC, IFAPA Alameda del Obispo, Cordoba, Spain) [37], whose oils display contrasting phenolic contents, were studied: Picual, Menya, Shengeh, Piñonera, Klon, Dokkar, and Abou Kanani. Olive trees were grown in the same agroclimatic and irrigation conditions as previously described [26] and fruits were harvested at four ripening stages: I, green fruits harvested 16–19 weeks after flowering (WAF); II, yellow-green fruits (22–25 WAF); III, turning fruits (28–31 WAF); and IV, fully ripe fruits (35–40 WAF).

### 3.2. Identification of Phenylacetaldehyde Reductase Full-Length cDNA

Identification of putative *PAR* genes that might be involved in the biosynthesis of tyrosol and hydroxytyrosol in olive was carried out with the help of the Blast2GO software [29] using the sequences of two PARs from *Rhodiola rosea* [25], Rr4HPAR1 (GenBank MF674524) and Rr4HPAR2 (GenBank MF674525), as the query against a transcriptome generated from the same olive cultivars in this study at the fruit ripening stages II and IV [26] annotated against the olive genome data base (OE6.OLIVEFAT, https://denovo.cnag.cat/olive_data, (access on 27 November 2020)).

### 3.3. OePAR Genes Cloning, Heterologous Protein Expression and Purification

The coding sequences of the selected candidate genes from olive (*OePAR1.1*, OE6A039209 and *OePAR1.2*, OE6A025790) were synthesized with *E. coli* codon optimization (GenScript) and cloned into pET-28a (+)-TEV vector as *Nde*I-*Xho*I fragment (*OePAR1.1 and OePAR1.2* constructs). BL21(DE3) lacIq *E. coli* cells containing *OePAR 1.1 or OePAR1.2* constructs were grown at 37 °C in Luria Bertani media (LB), NaCl 0.5 M, with OD_600_ of 0.6, induced with 0.4 mM isopropyl-b-D-thiogalactoside (IPTG), and allowed to grow for an additional 22 h at 19 °C. Cells were harvested by centrifugation, washed with PBS buffer (137 mM NaCl, 2.7 mM KCl, 10 mM Na_2_HPO4, and 1.8 mM KH_2_PO4), resuspended in lysis buffer (50 mM Tris-HCl, pH 8.0, 0.5 M NaCl, 60 mM imidazole, 0.5 mM DTT, 1 mM PMSF, a protease inhibitor cocktail, Sigma-Aldrich, St. Louis, MO, USA), and lysed by sonication. The resulting crude protein lysate was clarified by centrifugation prior to nickel–sepharose chromatographic purification with His-SpinTrap affinity columns (GE Healthcare, Chicago, IL, USA). After loading the clarified lysate, His-tagged recombinant protein-bound Ni resin was washed with six column volumes of wash buffer (50 mM Tris-HCl, pH 7.4, 0.5 M NaCl, 60 mM imidazole, and 0.5 mM DTT) and eluted with two column volume of elution buffer (50 mM Tris, pH 7.4, 0.5 M NaCl, 500 mM imidazole and 0,5 mM DTT). Imidazole was removed on a PD-10 desalting column (Sephadex G-25, GE Healthcare, Chicago, IL, USA). After dialysis, protein solution was concentrated using a Vivaspin centrifugal concentrator (MWCO 30 kDa, Merck, Darmstadt, Germany) and stored at 4 °C in storage buffer (20 mM Tri-HCl, pH 8.0, 25 mM NaCl, 0,5 mm DTT, and 10% glycerol). Purity of the recombinant protein was assessed by SDS-PAGE and densitometric analysis (ChemiDoc Imaging System, Biorad, Hercules, CA, USA). Concentration of the purified recombinant protein was determined by Bio-Rad protein assay kit using bovine serum albumin as a standard.

### 3.4. Total RNA Extraction and Gene Expression Analysis

Total RNA extraction from olive mesocarp tissues was performed using the Spectrum Plant Total RNA Kit (Sigma-Aldrich, St. Louis, MO, USA) according to the supplier’s instructions. The corresponding cDNAs were synthesized using the Ready-To-Go T-Primed First Strand Kit (Amersham Bioscience, Roosendaal, The Netherlands). The cDNAs were subjected to RT-QPCR with specific pair of primers for two olive phenolic aldehyde reductase (*OePAR1.1 and OePAR1.2*) (Table S5) and using SYBR Green I (SsoAdvanced^TM^ Universal SYBR^®^ Green Supermix, Biorad, Hercules, CA, USA) in a CFX96 Touch System (Biorad, Hercules, CA, USA) to monitor the resulting fluorescence. The reaction mixture was heated to 95 °C for 30 s before subjecting it to 40 PCR cycles consisting of: 95 °C for 15 s; 54 °C for 15 s; and 60 °C for 15 s. Efficiency curves were drawn up for each pair of primers using sequential dilutions of cDNA. The Pfaffl method [38] was applied using the BioRad CFX Maestro 1.0 Software (Biorad, Hercules, CA, USA) and excel sheet to calculate comparative expression levels between samples. Pfaffl formula applied was E^dCT(gen)^/[(E^dCt(Ref1)^xE^dCT(Ref2)^]^1/2^, for E = efficiency; dCT = Ct(calibrator)-Ct(gen); calibrator= Ct to which all the Cts are relativized. Two olive genes, elongation factor-1-alpha (*OeEF1α*) and glyceraldehyde-3-phosphate dehydrogenase (*OeGAPDH*) (Olive genome Data Base annotation number OE6A045598 and OE6A105640, https://denovo.cnag.cat/olive_data, access on 27 November 2020) were selected as reference genes according to previous validation studies [26] with the GeNorm software included in the BioRad CFX Maestro 1.0 software. Specific pair of primers for these reference genes and *OePAR1.1* and *OePAR1.2* are described in Table S4. Three biological and two technical replicates were obtained from each cultivar and maturity stage. Each biological replicate consisted of an independent extract from pooled mesocarp tissue from olives harvested from two different trees. Statistical significance was set at a level of *p* < 0.05 (Student’s *t*-test).

### 3.5. Sequence Alignment and Phylogenetic Analysis

The multiple sequence alignments of plant PAR amino acid sequences were calculated using the ClustalX program (https://clustalx.software.informer.com, access on 15 February 2021) and displayed with GeneDoc (https://genedoc.software.informer.com, access on 15 February 2021).

The phylogenetic tree analysis was performed on the Phylogeny.fr platform [39] and comprised the sequences alignment with MUSCLE (v3.8.31) [40] and removal of ambiguous regions with Gblocks (v0.91b) [41]. The phylogenetic tree was reconstructed using the maximum likelihood method implemented in the PhyML program (v3.1/3.0 aLRT) [42]. Reliability for internal branch was assessed using the aLRT test (SH-Like) [43]. Graphical representation and edition of the phylogenetic tree were performed with TreeDyn (v198.3) [44].

The conserved domains in the deduced amino acid sequences were analyzed using the NCBI Conserved Domain Search (https://www.ncbi.nlm.nih.gov/cdd/, access on 15 February 2021) and Pfam software (http://pfam.sanger.ac.uk/, access on 15 February 2021). Accession numbers of the different PARs included in the analysis are listed as Supplementary Data (Table S2).

The expected isoelectric point (pI) and molecular mass of the studied proteins were calculated with ExPASy Compute pI/Mw https://web.expasy.org/compute_pi/, access on 1 September 2019) [45]. The subcellular localizations of the proteins were predicted using DeepLoc1.0 on-line tool (http://www.cbs.dtu.dk/services/DeepLoc/, access on 1 September 2019) [31] and the presence of some signal peptide at the N-terminus was assessed with TargetP2.0 (http://www.cbs.dtu.dk/services/TargetP/, access on 1 September 2019) [32].

### 3.6. OePAR Activity Assay

The PAR enzyme assays were performed in 150 µL of reaction buffer (50 mM Tris-HCl pH 8.0, 5 mM NADPH) containing 0.5–2.0 µg of the recombinant enzymes OePAR1.1 or OePAR1.2 and using 3,4-DHPAA 2 mM as substrate (Cayman chemicals, Ann Arbor, MI, USA). The mixture was incubated at 30 °C for 5 min and then the reaction was terminated by the addition of 150 µL 0.8 M formic acid. The reaction mixture was centrifuged and filtered (0.45 µm) and the supernatant was analyzed by HPLC using the same equipment and chromatographic conditions described below for the analysis of phenolic compounds.

The optimum pH was determined using sodium acetate, phosphate, and borate buffers (50 mM) in the standard assay. The optimum temperature was determined using a temperature interval of 20–70 °C in the standard assay. Thermal stability was determined under standard assay conditions after incubation of purified preparation at different temperatures for 60 min.

### 3.7. Olive Oil Extraction

Olive oil was extracted using an Abencor analyzer (Comercial Abengoa, S.A., Seville, Spain) that simulates the industrial process of VOO production on a laboratory scale. Processing parameters have been precisely described in a previous study [26].

### 3.8. Extraction and Analysis of Fruit and VOO Phenolic Compounds

Fruit phenolic compounds were extracted according to a previously developed protocol [46]. Longitudinal pieces of mesocarp tissue were cut from 20 olive fruits and kept at 4 °C for 72 h in dimethyl sulfoxide (6 mL/g of fruit), containing syringic acid (24 mg/mL) as the internal standard. The extracts were filtered through a 0.45-µm mesh nylon and kept at −20 °C until HPLC analysis.

VOO phenolic compounds were isolated by solid phase extraction (SPE) on a diol-bonded phase cartridge (Supelco, Bellefonte, PA, USA) based on a previously described method [47] using *p*-hydroxyphenyl-acetic and *o-*coumaric acids as internal standards. Response factors were calculated for each phenolic compound as described previously [4].

Phenolic compounds from fruits and oils were analyzed by HPLC on a Beckman Coulter liquid chromatography system equipped with a System Gold 168 detector, a solvent module 126, an autosampler module 508, and a Waters column heater module as described previously [4] using a Superspher RP 18 column (4.6 mm i.d. × 250 mm, particle size 4 µm: Dr Maisch GmbH, Ammerbuch, Germany) at flow rate 1 mL min^−1^ and a temperature of 35 °C. Identification of compounds was confirmed by HPLC/ESI-qTOF-HRMS on a liquid chromatograph Dionex Ultimate 3000 RS U-HPLC liquid chromatograph system (Thermo Fisher Scientific, Waltham, MA, USA) equipped with a similar column and elution program. Mass spectra were acquired in MS fullscan mode and data were processed using TargetAnalysis 1.2 software (Bruker Daltonics, Bremen, Germany).

## 4. Conclusions

Despite the recent interest in the secoiridoid compounds derived from hydroxytyrosol due to their health-promoting properties, the biochemical pathways involved in the formation of these compounds in olive are not yet completely clarified. The integration of the metabolic and gene expression data sets has made it possible to identify some potentially decisive genes in this synthesis pathway, whose functional characterization makes it possible to evaluate their role in the metabolism of phenolic compounds. Thus, we have identified and characterized two highly specific phenyl acetaldehyde reductase enzymes, OePAR1.1 and OePAR1.2, which catalyze the last step in the synthesis of hydroxytyrosol in olive. In this way, the hydroxytyrosol synthesis from L-DOPA is completed, which comprises the participation of only two enzymes, the OeAAS previously reported and the OePARs characterized in this study. The joint analysis of the gene expression data of PAR and AAS and the correlation with the contents of hydroxytyrosol derivatives in olive suggest that the limiting step in the synthesis of hydroxytyrosol from L-DOPA is the formation of 3,4-DHPAA, the substrate of PAR proteins. Thus, given the proved functional properties of hydroxytyrosol, the olive *PAR* and, especially, *AAS* genes could be good candidates in the framework of the search for molecular markers for olive breeding programs for the identification at the seedling stage of olive genotypes with the capacity to produce oils with higher levels of hydroxytyrosol derivatives.

## Supplementary Materials

The following are available online at https://www.mdpi.com/article/10.3390/plants10071268/s1. Figure S1: Multiple sequence alignment of the six putative olive PAR proteins. Figure S2: Recombinant OePAR proteins induction. Figure S3: Effect of pH on OePAR proteins activity. Figure S4: Effect of temperature on OePAR proteins activity. Figure S5: Thermal inactivation kinetics. Figure S6: Kinetic parameters. Table S1: Expression level and homologies of six putative PAR transcripts selected. Table S2: Accession numbers of genes used for phylogenetic analysis. Table S3: Phenolic composition of fruits from Picual, Menya, Shengeh, Piñonera cultivars harvested along ripening. Table S4: Phenolic composition of virgin olive oils. Table S5: Real time-quantitative PCR primers used in this work.

## References

[1] Obied H.K., Prenzler P.D., Ryan D., Servili M., Taticchi A., Esposto S., Robards K. (2008). Biosynthesis and biotransformations of phenol-conjugated oleosidic secoiridoids from *Olea europaea* L.. Nat. Prod. Rep..

[2] Romero-Segura C., García-Rodríguez R., Sánchez-Ortiz A., Sanz C., Pérez A.G. (2012). The role of olive beta-glucosidase in shaping the phenolic profile of virgin olive oil. Food Res. Int..

[3] Romero-Segura C., García-Rodríguez R., Sanz C., Pérez A.G. (2011). Virgin olive phenolic profile as a result of the anabolic and catabolic enzymes status in the olive fruit. Acta Hortic..

[4] García-Rodríguez R., Belaj A., Romero-Segura C., Sanz C., Pérez A.G. (2017). Exploration of genetic resources to improve the functional quality of virgin olive oil. J. Funct. Foods.

[5] Lozano-Castellón J., López-Yerena A., de Alvarenga J.F.R., del Castillo-Alba J.R., Vallverdú-Queralta A., Escribano-Ferrer E., Lamuela-Raventósa R.M. (2019). Health-promoting properties of oleocanthal and oleacein: Two secoiridoids from extra-virgin olive oil. Crit. Rev. Food Sci. Nutr..

[6] Andrewes P., Busch J.L.H.C., de Joode T., Groenewegen A., Alexandre H. (2003). Sensory properties of virgin olive oil polyphenols: Identification of deacetoxy-ligstroside glycon as a key contributor to pungency. J. Agric. Food Chem..

[7] Covas M.-I., de la Torre R., Fitó M. (2015). Virgin olive oil: A key food for cardiovascular risk protection. Br. J. Nutr..

[8] Mateos R., Pereira-Caro G., Saha S., Cert R., Redondo-Horcajo M., Bravo L., Kroon P.A. (2011). Acetylation of hydroxytyrosol enhances its transport across differentiated Caco-2 cell monolayers. Food Chem..

[9] Bernardini E., Visioli F. (2017). High quality, good health: The case for olive oil. Eur. J. Lipid Sci. Technol..

[10] Angeloni C., Malaguti M., Barbalace M.C., Hrelia S. (2017). Bioactivity of Olive Oil Phenols in Neuroprotection. Int. J. Mol. Sci..

[11] European Commission (2012). Commission Regulation (EU) No 1018/2013 amending Regulation (EU) No 432/2012 establishing a list of permitted health claims made on foods other than those referring to the reduction of disease risk and to children’s development and heal. Off. J. Eur. Union L.

[12] Costanzo P., Bonacci S., Cariati L., Nardi M., Oliverio M., Procopio A. (2018). Simple and efficient sustainable semi-synthesis of oleacein [2-(3,4-hydroxyphenyl) ethyl (3S,4E)-4-formyl-3-(2-oxoethyl)hex-4-enoate] as potential additive for edible oils. Food Chem..

[13] Pérez A.G., León L., Sanz C., De la Rosa R. (2018). Fruit Phenolic Profiling: A New Selection Criterion in Olive Breeding Programs. Front. Plant Sci..

[14] García-Vico L., Sánchez R., Fernández G., Sanz C., Pérez A.G. (2021). Study of the olive β-glucosidase gene family putatively involved in the synthesis of phenolic compounds of virgin olive oil. J. Sci. Food Agric..

[15] Alagna F., Mariotti R., Panara F., Caporali S., Urbani S., Veneziani G., Esposto S., Taticchi A., Rosati A., Rao R. (2012). Olive phenolic compounds: Metabolic and transcriptional profiling during fruit development. BMC Plant Biol..

[16] Mougiou N., Trikkab F., Trantasc E., Ververidisc F., Makrisb A., Argirioub A., Vlachonasiosa K.E. (2018). Expression of hydroxytyrosol and oleuropein biosynthetic genes are correlated with metabolite accumulation during fruit development in olive, Olea europaea, cv. Koroneiki. Plant. Physiol. Biochem..

[17] Guodong R., Jianguo Z., Xiaoxia L., Ying L. (2019). Identification of putative genes for polyphenol biosynthesis in olive fruits and leaves using full-length transcriptome sequencing. Food Chem..

[18] Alagna F., Geu-Flores F., Kries H., Panara F., Baldoni L., O’Connor S., Osbourn A. (2016). Identification and Characterization of the Iridoid Synthase Involved in Oleuropein Biosynthesis in Olive (*Olea europaea*) Fruits. J. Biol. Chem..

[19] Volk J., Sarafeddinov A., Unver T., Marx S., Tretzel J., Zotzel J., Warzecha H. (2019). Two novel methylesterases from *Olea europaea* contribute to the catabolism of oleoside-type secoiridoid esters. Planta.

[20] Velázquez-Palmero D., Romero-Segura C., García-Rodríguez R., Hernández M.L., Vaistij F.E., Graham I.A., Pérez A.G., Martínez-Rivas J.M. (2017). An oleuropein β-glucosidase from olive fruit is involved in determining the phenolic composition of virgin olive oil. Front. Plant Sci..

[21] Hazelwood A., Daran J.M., van Maris A.J.A., Pronk J.T., Dickinson J.R. (2008). The Ehrlich pathway for fusel alcohol production: A century of research on *Saccharomyces cerevisiae* metabolism. Appl. Environ. Microbiol..

[22] Li C., Jia P., Bai Y., Fan T., Zheng X., Cai Y.E. (2019). Efficient synthesis of hydroxytyrosol from l-3,4-dihydroxyphenylalanine using engineered *escherichia coli* whole cells. J. Agric. Food Chem..

[23] Muñiz-Calvo S., Bisquet R., Puig S., Guillamon J.M. (2020). Overproduction of hydroxytyrosol in *Sacharomyces cerevisiae* by heterologous overexpression of the *E.Coli* 4-hydroxyphenylacetate 3 monooxygenase. Food Chem..

[24] Lan X., Chang K., Zeng L., Liu X., Qiu F., Zheng W., Quan H., Liao Z., Chen M., Huang W. (2013). Engineering salidroside biosynthetic pathway in hairy root cultures of *Rhodiola crenulata* based on metabolic characterization of tyrosine decarboxylase. PLoS ONE.

[25] Torrens-Spence M.P., Pluskal T., Li F.-S., Carballo V., Weng J.-K. (2018). Complete Pathway Elucidation and Heterologous Reconstitution of Rhodiola Salidroside Biosynthesis. Mol. Plant..

[26] Sánchez R., García-Vico L., Sanz C., Pérez A. (2019). An Aromatic Aldehyde Synthase Controls the Synthesis of Hydroxytyrosol Derivatives Present in Virgin Olive Oil. Antioxidants.

[27] Tieman D.M., Loucas H.M., Kim J.Y., Clark D.G., Klee H.J. (2007). Tomato phenylacetaldehyde reductases catalyze the last step in the synthesis of the aroma volatile 2-phenylethanol. Phytochemistry.

[28] Chen X., Kobayashi H., Sakai M., Hirata H., Asai T., Ohnishi T., Baldermann S., Watanabe N. (2011). Functional characterization of rose phenylacetaldehyde reductase (PAR), an enzyme involved in the biosynthesis of the scent compound 2-phenylethanol. J. Plant Physiol..

[29] Conesa A., Götz S., García-Gómez J.M., Terol J., Talón M., Robles M. (2005). Blast2GO: A universal tool for annotation, visualization and analysis in functional genomics research. Bioinformatics.

[30] Kim S.-J., Kim M.-R., Bedgar D.L., Moinuddin S.G.A., Cardenas C.L., Davin L.B., Kang C., Lewis N.G. (2004). Functional reclassification of the putative cinnamyl alcohol dehydrogenase multigene family in Arabidopsis. Proc. Natl. Acad. Sci. USA.

[31] Almagro-Armenteros J., Sønderby C., Sønderby S., Nielsen H., Winther O. (2017). DeepLoc: Prediction of protein subcellular localization using deep learning. Bioinformatics.

[32] Armenteros J.A., Salvatore M., Emanuelsson O., Winther O., von Heijne G., Elofsson A., Nielsen H. (2019). Detecting sequence signals in targeting peptides using deep learning. Life Sci. Alliance.

[33] Günther J., Lackus N.D., Schmidt A., Huber M., Stödtler H.-J., Reichelt M., Gershenzon J., Köllner T.G. (2019). Separate pathways contribute to the herbivore-induced formation of 2-phenylethanol in poplar. Plant Physiol..

[34] Zhou Y., Zhang L., Gui J., Dong F., Cheng F., Mei X., Zhang L., Li Y., Su X., Baldermann S. (2015). Molecular Cloning and Characterization of a Short-Chain Dehydrogenase Showing Activity with Volatile Compounds Isolated from *Camellia sinensis*. Plant Mol. Biol. Rep..

[35] Saimaru H., Orihara Y. (2010). Biosynthesis of acteoside in cultured cells of *Olea europaea*. J. Nat. Med..

[36] Ansari E., Karami A., Ebrahimie E. (2019). Isolation of 2-phenylethanol biosynthesis related gene and developmental patterns of emission of scent compounds in Persian musk rose (*Rosa moschata Herrm*.). Biocatal. Agric. Biotechnol..

[37] Belaj A., Domínguez-Garcia M.C., Atienza S.G., Martin-Urdiroz N., de la Rosa R., Satovic Z., del Río C. (2012). Developing a core collection of olive (*Olea europaea* L.) based on molecular markers (DArTs, SSRs, SNPs) and agronomic traits. Tree Genet. Genomes.

[38] Pfaffl M.W. (2001). A new mathematical model for relative quantification in real-time RT-PCR. Nucleic Acids Res..

[39] Dereeper A., Guignon V., Blanc G., Audic S., Buffet S., Chevenet F., Dufayard J.F., Guindon S., Lefort V., Lescot M. (2008). Phylogeny.fr: Robust phylogenetic analysis for the non-specialist. Nucleic Acids Res..

[40] Edgar R.C. (2004). MUSCLE: Multiple sequence alignment with high accuracy and high throughput. Nucleic Acids Res..

[41] Castresana J. (2000). Selection of conserved blocks from multiple alignments for their use in phylogenetic analysis. Mol. Biol. Evol..

[42] Guindon S., Gascuel O. (2003). A simple, fast, and accurate algorithm to estimate large phylogenies by maximum likelihood. Syst. Biol..

[43] Anisimova M., Gascuel O. (2006). Approximate likelihood ratio test for branchs: A fast, accurate and powerful alternative. Syst. Biol..

[44] Chevenet F., Brun C., Banuls A.L., Jacq B., Chisten R. (2006). TreeDyn: Towards dynamic graphics and annotations for analyses of trees. BMC Bioinform..

[45] Gasteiger E., Hoogland C., Gattiker A., Duvaud S., Wilkins M.R., Appel R.D., Bairoch A., Walker J.M. (2005). Protein Identification and Analysis Tools on the ExPASy Server. The Proteomics Protocols Handbook.

[46] Fernández G., García-Vico L., Sanz C., Pérez A.G. (2020). Optimization of a simplified method for fruit phenolic extraction and analysis to be used in olive breeding. Acta Hortic..

[47] Mateos R., Espartero J., Trujillo M., Ríos J., León-Camacho M., Alcudia F., Cert A. (2001). Determination of Phenols, Flavones, and Lignans in Virgin Olive Oils by Solid-Phase Extraction and High-Performance Liquid Chromatography with Diode Array Ultraviolet Detection. J. Agric. Food Chem..

