# Supplementary material for: Identification and Functional Characterization of Genes Encoding Phenylacetaldehyde Reductases That Catalyze the Last Step in the Biosynthesis of Hydroxytyrosol in Olive
Source: Plants (Basel). 2021 Jun 22;10(7):1268. doi: 10.3390/plants10071268 (PMC8309162; doi:10.3390/plants10071268)
Supplement: Supplementary file 1 [file plants-10-01268-s001.zip › plants-1254401-supplementary.pdf]

Supplementary Figures and tables

Table S1: Putative PAR transcripts selected. Identities and mean expression levels in the transcriptome of seven selected olive cultivars [26].

Transcript (Olive genome ID)	e-value	Homology	Identity (%)	Mean expression level in transcriptome (FPKM)
Oe6A039209	0	Rr4HPAR1	90.0	10666
Oe6A025790	0	Rr4HPAR1	88.7	81045
Oe6A018558	0	Rr4HPAR1	88.3	414
Oe6A068874	1×10^{-147}	Rr4HPAR2	83.0	576
Oe6A030751	1×10^{-148}	Rr4HPAR2	82.8	860
Oe6A074153	1×10^{-142}	Rr4HPAR2	79.0	54

Table S2: Accession numbers of genes used for phylogenetic analysis.

**Identifier**	**Gene Bank Accession**	**Organism**
EgCCR	O04877	*Eucalyptus gunni*
EgCAD1	O04391	*Eucalyptus gunni*
ZmCCR	CAA66707.1	*Zea mays*
ZmDFR	P51108.1	*Zea mays*
VuCPRD14	P93700	*Vigna unguiculata*
VrERE	AAD53967.1	*Vigna radiata*
DcDFR	P51104.1	*Dianthus caryophyllus*
VvDFR	P51110.1	*Vitis vinifera*
VvDFR	P51110.1	*Vitis vinifera*
LeADH	ABR15770	*Solanum lycopersicum*
LePAR1	ABR15768.1	*Solanum lycopersicum*
LePAR2	ABR15769.1	*Solanum lycopersicum*
PtAAR1	QBL52492.1	*Populus trichocarpa*
PtAAR2	QBL52491.1	*Populus trichocarpa*
PtAAR3	QBL52490.1	*Populus trichocarpa*
PtPAR1	QBL52487.1	*Populus trichocarpa*
PtPAR2	QBL52486.1	*Populus trichocarpa*
OE6A018558		*Olea europaea*
OE6A030751		*Olea europaea*
OE6A068874		*Olea europaea*
OE6A074153		*Olea europaea*
OePAR1.1	MW038826	*Olea europaea*
OePAR1.2	MW038827	*Olea europaea*
Rr4HPAR1,	AUI41113	*Rhodiola rosea*
Rr4HPAR2,	AUI41114	*Rhodiola rosea*
SbDFR	P93776	*Sorghum bicolor*
PhDFR	P14720.2	*Petunia hybrida*
RhDFR	BAA12723.1	*Rosa hybrid*

Figure S1. Multiple sequence alignment of the six putative olive PAR proteins with the PAR proteins fully characterized in *Rhodiola rosea*, *Populus trichocarpa* and *Solanum lycopersicum*. The multiple sequence alignments of plant PAR amino acid sequences were calculated using the ClustalX program (https://clustalx.software.informer.com) and displayed with GeneDoc (https://genedoc.software.informer.com). The highly conserved residues are shaded. Active site motif YXXXK is squared in red and amino acids forming the canonical catalytic triad, Ser130-Tyr165-Lys169, are indicated with a green triangle (Rr4HPAR1 numbering). NADP-binding motif TGXXGXX(GA) is squared in blue and amino acids involved in cofactor binding are indicated with orange triangle. Accession numbers are given in Table S2.

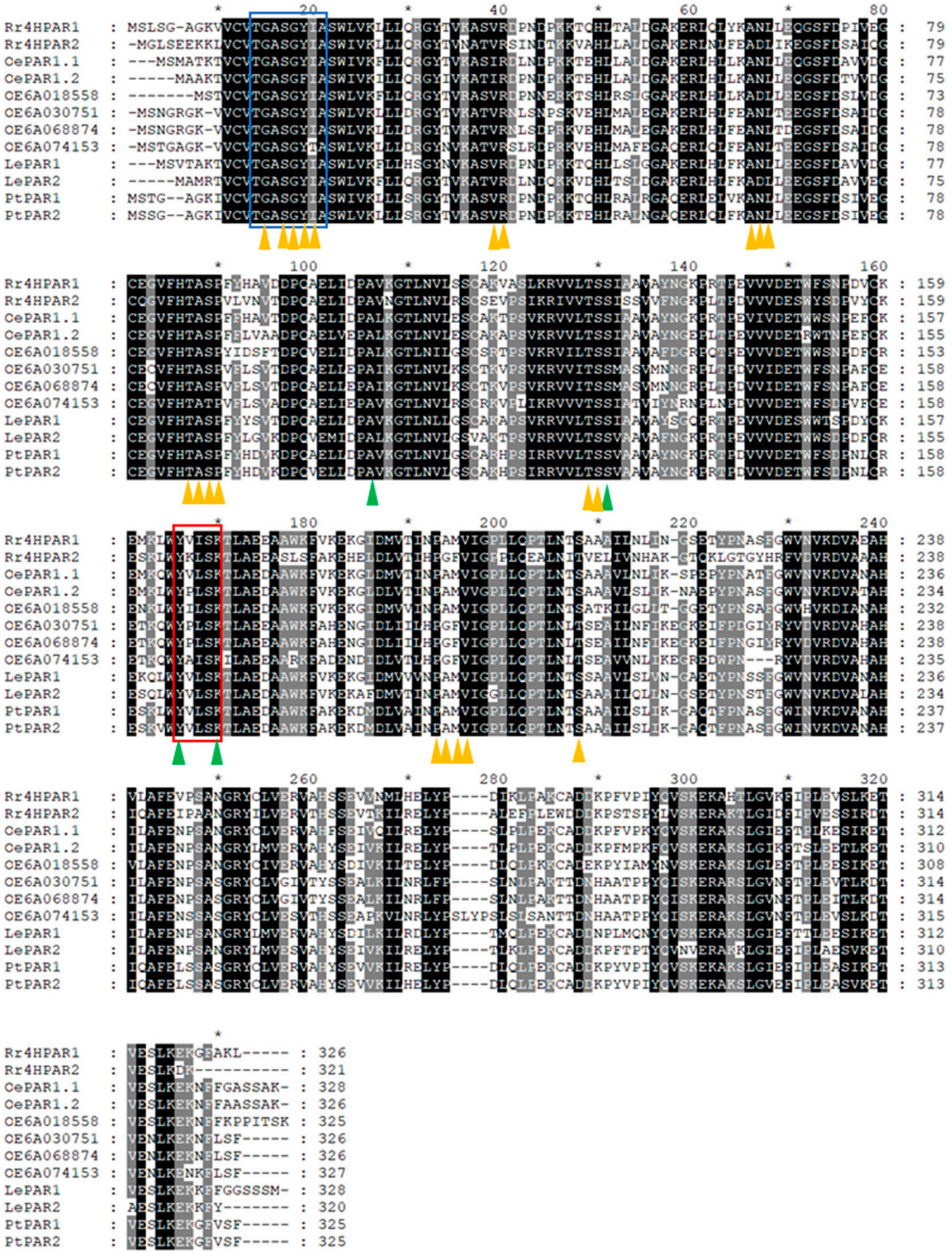

Figure S2. Induction of recombinant OePAR proteins in *Escherichia coli***.** SDS-PAGE followed by a Coomassie blue staining showing OePARs presence in *E. coli* culture total (T) and soluble (S) fractions. Arrow shows OePAR protein at the expected molecular weight (MW).

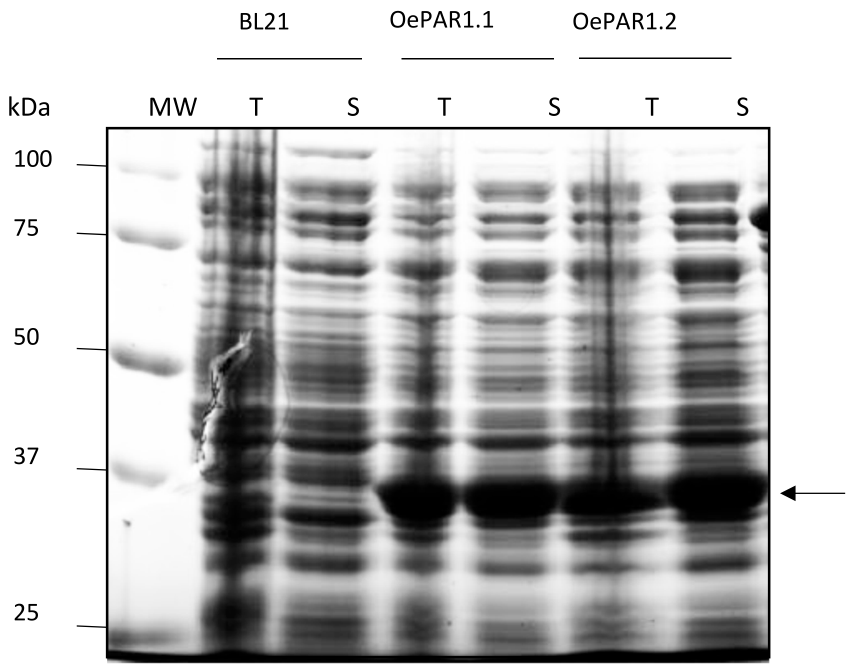

Figure S3. Effect of pH on the activity of OePAR1.1 and OePAR1.2 proteins.

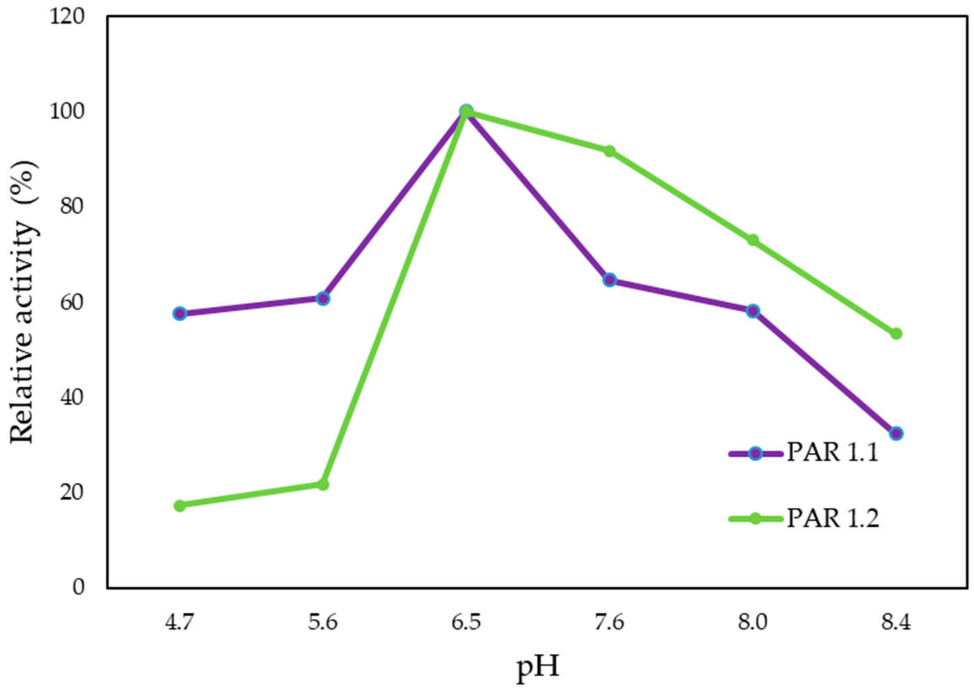

Figure S4: Effect of temperature on the activity of OePAR1.1 and OePAR1.2 proteins

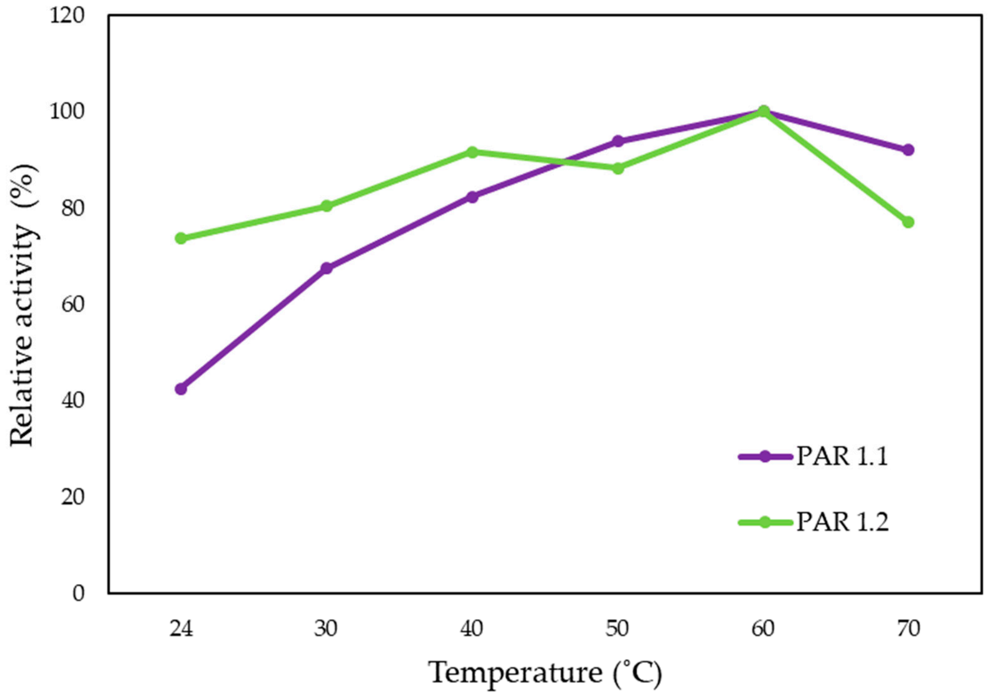

Figure ~~S3~~S5**.** Thermal inactivation kinetics of OePAR1.1 and OePAR1.2 proteins.

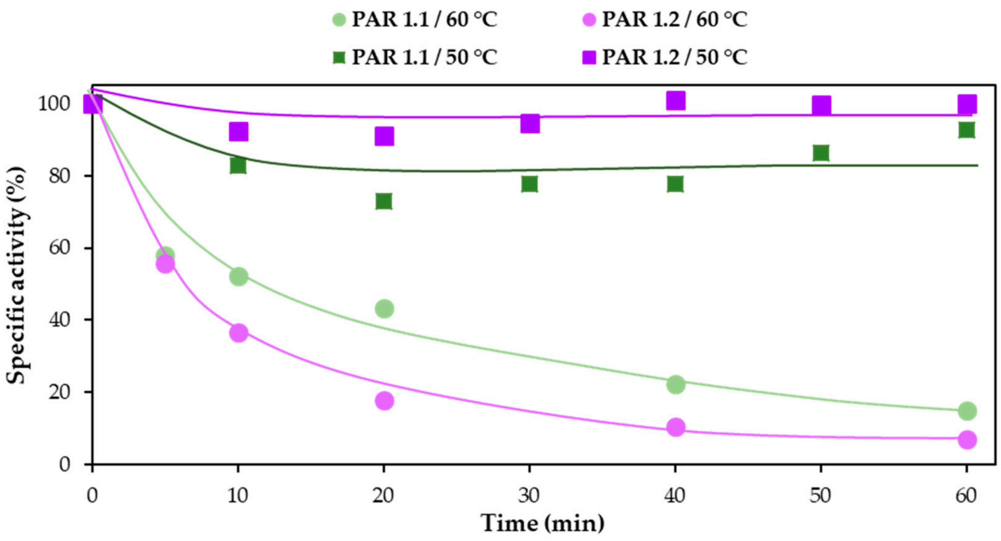

Figure ~~S4~~S6**:** Kinetic parameters calculated for OePAR1.1 and OePAR1.2

PAR 1.1

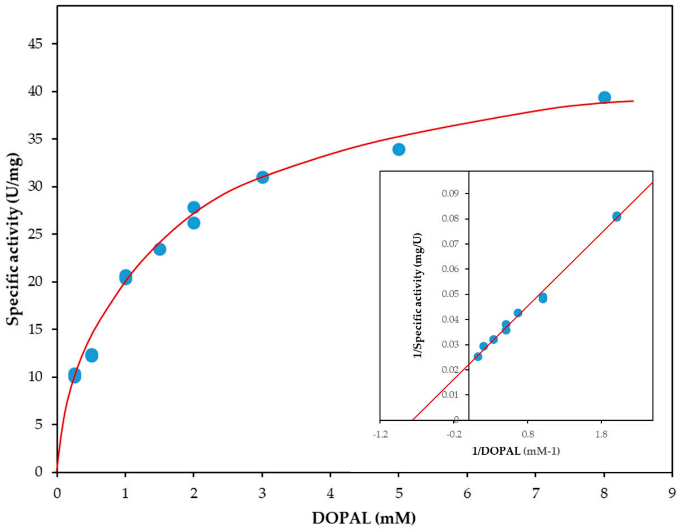
Specific activity (U/mg)
DOPAL (mM)
1/Specific activity (mg/U)
1/DOPAL (mM-1)

PAR1.2

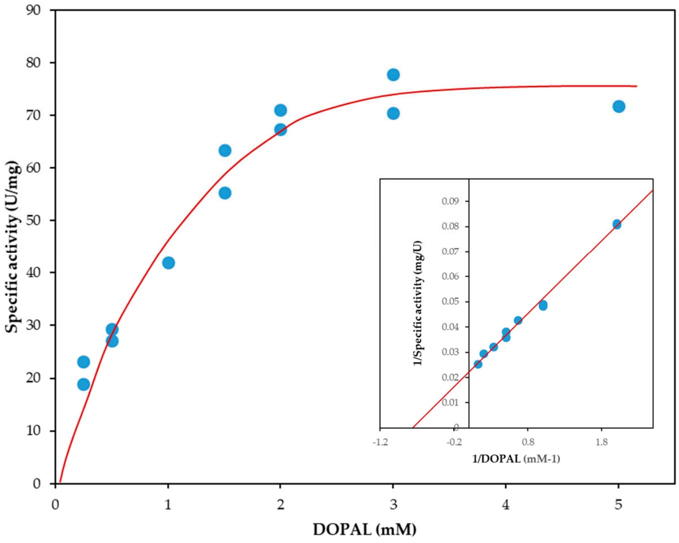
Specific activity (U/mg)
DOPAL (mM)
1/Specific activity (mg/U)
1/DOPAL (mM-1)

Table S3. Phenolic composition of olive fruits from cultivars Picual, Menya, Shengeh and Piñonera along ripening.

	Picual											
Phenolics (μmol/ g)	Stage I			Stage II			Stage III			Stage IV		
Hydroxytyrosol-G	1.52	±	0.03	1.52	±	0.12	0.90	±	0.01	3.09	±	0.10
Tyrosol-G	0.23	±	0.01	0.23	±	0.01	0.15	±	0.00	0.32	±	0.01
Demethyloleuropein	0.46	±	0.07	0.16	±	0.04	0.27	±	0.00	0.03	±	0.00
Demethyligstroside	0.31	±	0.00	0.07	±	0.03	0.07	±	0.00	0.04	±	0.01
Oleuropein	57.11	±	2.73	51.27	±	1.86	34.80	±	0.12	14.43	±	0.16
Ligstroside	4.80	±	0.46	4.22	±	0.27	2.53	±	0.04	1.44	±	0.01
Verbascoside	2.26	±	0.41	3.12	±	0.28	2.01	±	0.11	2.91	±	0.05
Luteolin -7-G	1.51	±	0.25	1.10	±	0.15	1.01	±	0.02	0.40	±	0.03
total phenolics	68.20	±	3.96	61.68	±	2.77	41.75	±	0.31	22.66	±	0.36
	Menya											
Phenolics (μmol/ g)	Stage I			Stage II			Stage III			Stage IV		
Hydroxytyrosol-G	1.38	±	0.09	1.81	±	0.53	1.47	±	0.09	1.41	±	0.04
Tyrosol-G	0.20	±	0.02	0.30	±	0.09	0.17	±	0.00	0.33	±	0.00
Demethyloleuropein	0.51	±	0.00	0.24	±	0.04	2.79	±	0.73	15.89	±	0.79
Demethyligstroside	0.07	±	0.00	0.08	±	0.01	0.06	±	0.01	0.03	±	0.01
Oleuropein	61.58	±	5.58	55.26	±	3.03	44.94	±	1.72	5.06	±	0.10
Ligstroside	5.33	±	0.65	6.32	±	0.15	6.00	±	0.47	0.71	±	0.02
Verbascoside	0.18	±	0.00	0.20	±	0.07	0.40	±	0.07	0.52	±	0.00
Luteolin -7-G	0.11	±	0.00	0.33	±	0.05	0.27	±	0.06	0.08	±	0.01
total phenolics	69.36	±	6.35	64.53	±	3.96	56.12	±	3.17	24.03	±	0.98
	Shengeh											
Phenolics (μmol/ g)	Stage I			Stage II			Stage III			Stage IV		
Hydroxytyrosol-G	0.63	±	0.05	1.54	±	0.01	2.17	±	0.02	2.54	±	0.29
Tyrosol-G	0.05	±	0.01	0.09	±	0.00	0.28	±	0.00	0.35	±	0.03
Demethyloleuropein	0.28	±	0.01	0.32	±	0.03	0.05	±	0.00	0.40	±	0.01
Demethyligstroside	0.08	±	0.00	0.12	±	0.00	0.09	±	0.10	0.05	±	0.00
Oleuropein	21.84	±	1.15	12.25	±	0.61	7.04	±	0.57	7.97	±	0.00
Ligstroside	2.34	±	0.12	1.92	±	0.18	1.38	±	0.09	1.25	±	0.00
Verbascoside	0.02	±	0.00	0.16	±	0.01	0.07	±	0.03	0.19	±	0.02
Luteolin -7-G	0.33	±	0.04	0.36	±	0.06	0.24	±	0.02	0.18	±	0.02
total phenolics	25.57	±	1.38	16.76	±	0.91	11.32	±	0.83	12.92	±	0.38
	Piñonera											
Phenolics (μmol/ g)	Stage I			Stage II			Stage III			Stage IV		
Hydroxytyrosol-G	1.09	±	0.25	0.94	±	0.24	2.46	±	0.64	2.18	±	0.08
Tyrosol-G	0.32	±	0.16	0.21	±	0.05	0.35	±	0.09	0.35	±	0.00
Demethyloleuropein	0.42	±	0.05	0.29	±	0.06	3.24	±	1.58	15.80	±	0.61
Demethyligstroside	0.44	±	0.19	0.03	±	0.00	0.02	±	0.02	0.01	±	0.01
Oleuropein	46.41	±	4.36	36.57	±	0.13	29.22	±	3.66	4.70	±	0.03
Ligstroside	6.41	±	2.31	4.21	±	0.02	3.31	±	0.76	0.80	±	0.11
Verbascoside	1.85	±	0.15	2.10	±	0.65	2.16	±	0.12	2.17	±	0.08
Luteolin -7-G	0.33	±	0.08	0.20	±	0.10	0.38	±	0.08	0.17	±	0.02
total phenolics	57.25	±	7.55	44.55	±	1.25	41.14	±	6.93	26.17	±	0.94

(*) Data are mean contents and standard deviations from three different analyses.

Table S4. Phenolic composition of virgin olive oils obtained from Picual, Menya, Shengeh, Piñonera, Klon, Dokkar and Abou Kanani cultivars.

Olive cultivar<br>Phenolics (µg/g oil)	Picual III			Menya III			Shengeh III			Piñonera III			Klon III			Dokkar III			Abou Kanani III		
Hydroxytyrosol	1.5	±	0.32	1.1	±	0.1	1.0	±	0.1	1.0	±	0.1	0.3	±	0.2	1.7	±	1.5	0.5	±	0.2
Tyrosol	1.9	±	0.1	3.8	±	0.1	3.4	±	0.1	3.4	±	0.1	3.7	±	0.2	2.3	±	0.2	1.4	±	0.0
Vanillic acid	0.2	±	0.0	0.4	±	0.0	0.1	±	0.0	0.1	±	0.0	0.1	±	0.0	0.2	±	0.0	0.3	±	0.0
Vainillin	0.1	±	0.0	0.3	±	0.0	0.0	±	0.1	0.1	±	0.0	0.1	±	0.0	0.1	±	0.0	0.0	±	0.0
p-coumaric acid	0.9	±	0.0	0.2	±	0.0	0.5	±	0.5	0.5	±	0.1	2.2	±	2.6	0.2	±	0.0	0.3	±	0.0
Hydroxytyrosol ac.	0.3	±	0.3	2.4	±	0.2	3.8	±	30.0	3.8	±	0.0	5.2	±	0.2	3.2	±	0.4	2.5	±	0.1
3.4-DHPEA-EDA	39.2	±	1.4	121.3	±	1.2	59.6	±	4.4	59.6	±	4.4	165.7	±	5.0	158.2	±	13.1	124.2	±	5.6
p-HPEA-EDA	36.9	±	4.6	87.6	±	2.7	97.7	±	2.0	60.7	±	2.0	113.5	±	4.0	63.3	±	7.4	32.1	±	2.2
Pinoresinol	9.3	±	0.7	6.2	±	0.2	2.1	±	0.0	2.1	±	0.0	6.7	±	0.3	6.2	±	0.8	3.8	±	0.1
Cinnamic acid	1.3	±	0.3	2.2	±	0.2	0.4	±	0.0	0.4	±	0.0	2.2	±	0.3	0.6	±	0.1	0.4	±	0.0
Acetoxypinoresinol	32.1	±	5.9	47.7	±	3.8	4.4	±	0.0	4.4	±	0.0	49.3	±	0.6	10.9	±	1.4	24.3	±	0.2
3.4-DHPEA-EA	854.7	±	26.4	388.8	±	7.3	11.9	±	2.0	11.9	±	2.0	439.7	±	13.3	147.6	±	14.2	247.1	±	1.9
p-HPEA-EA	37.1	±	6.6	40.0	±	4.3	5.9	±	0.0	5.9	±	0.2	21.9	±	2.1	21.2	±	1.9	12.2	±	0.3
Feruli acid	0.0	±	0.0	0.0	±	0.0	0.1	±	0.0	0.1	±	0.0	0.1	±	0.2	0.0	±	0.0	0.1	±	0.0
Luteolin	8.3	±	0.1	1.5	±	0.0	2.3	±	0.0	2.3	±	0.0	7.2	±	0.1	0.7	±	0.1	7.8	±	0.1
Apigenin	1.9	±	0.1	0.2	±	0.0	0.3	±	0.0	0.3	±	0.0	0.5	±	0.7	0.3	±	0.0	2.5	±	0.1
total phenolics	1025.8	±	20.9	703.8	±	19.6	215.7	±	4.4	156.6	±	4.4	818.3	±	29.6	416.8	±	38.1	459.5	±	5.1
total *o*-phenolics	904.1	±	27.9	515.1	±	8.8	91.7	±	2.4.	78.6	±	2.4	618.1	±	18.8	311.4	±	26.3	382.0	±	3.5
total secoiridoids	967.9	±	25.8	637.7	±	15.4	138.1	±	4.6	138.1	±	4.6	740.7	±	24.3	390.3	±	36.6	415.6	±	5.5

(*) Data are mean contents and standard deviations from three different analyses.

Table S5. Real Time-Quantitative PCR primers used in this work.

Primer	Sequence (5′ → 3′)
qOePAR-Fc	GGAAACTGTCGAAAGCTTGAAGGAGAAG
qOePAR1.1-R	GAATTAAAGATGGGCACAAACACCTCA
qOePAR1.2-R	CCACTCTCTCAGTAGCATAACAAAGCTAG
qOeEF1α-F	TGCTCTATCTGGATTGCCATT
qOeEF1α-R	TCAAATGCCACCATGACTTC
qOeGAPDH-F	TGAGATGCTGCACAATGGTT
qOeGAPDH-R	CACGATAGGCTTACGCAACA
